# A Porcine Adenovirus with Low Human Seroprevalence Is a Promising Alternative Vaccine Vector to Human Adenovirus 5 in an H5N1 Virus Disease Model

**DOI:** 10.1371/journal.pone.0015301

**Published:** 2010-12-16

**Authors:** Ami Patel, Suresh Tikoo, Gary Kobinger

**Affiliations:** 1 National Microbiology Laboratory, Public Health Agency of Canada, Canadian Science Centre for Human and Animal Health, Winnipeg, Canada; 2 Department of Medical Microbiology, University of Manitoba, Winnipeg, Canada; 3 Vaccine and Infectious Disease Organization, University of Saskatchewan, Saskatoon, Canada; University of Rochester School of Medicine, United States of America

## Abstract

Human adenovirus 5 (AdHu5) vectors are robust vaccine platforms however the presence of naturally-acquired neutralizing antibodies may reduce vector efficacy and potential for re-administration. This study evaluates immune responses and protection following vaccination with a replication-incompetent porcine adenovirus 3 (PAV3) vector as an alternative vaccine to AdHu5 using an avian influenza H5N1 disease model. Vaccine efficacy was evaluated in BALB/c mice following vaccination with different doses of the PAV3 vector expressing an optimized A/Hanoi/30408/2005 H5N1 hemagglutinin antigen (PAV3-HA) and compared with an AdHu5-HA control. PAV3-HA rapidly generated antibody responses, with significant neutralizing antibody titers on day 21, and stronger cellular immune responses detected on day 8, compared to AdHu5-HA. The PAV3-HA vaccine, administered 8 days before challenge, demonstrated improved survival and lower virus load. Evaluation of long-term vaccine efficacy at 12 months post-vaccination showed better protection with the PAV3-HA than with the AdHu5-HA vaccine. Importantly, as opposed to AdHu5, PAV3 vector was not significantly neutralized by human antibodies pooled from over 10,000 individuals. Overall, PAV3-based vector is capable of mediating swift, strong immune responses and offer a promising alternative to AdHu5.

## Introduction

Experimental adenovirus-based vaccine vectors are promising alternatives to conventional vaccine platforms. In particular, human adenovirus serotype 5 (AdHu5) vectors are well-characterized and are being developed against several infectious disease models including influenza, hepatitis C, dengue and viral hemorrhagic fever viruses [1], [2], [3], [4]. Several candidates have demonstrated unique protective efficacy and can generate robust immune responses in both animal models and clinical trials [4], [5], [6], [7]. Pre-existing immunity against AdHu5 is, however, frequent in the human population and has been associated with undesirable clinical outcomes and the suspension of clinical trials [8], [9], [10]. One promising alternative is the development and evaluation of rare human, chimpanzee, or other mammalian adenovirus vectors with low seroprevalence in humans. A chimeric simian adenovirus 21 vector protected mice against lethal *Ebolavirus* challenge and generated robust T-cell responses against the glycoprotein in nonhuman primates [11]. A bovine adenovirus 3 (BAV3)–based vaccine previously demonstrated successful protection against avian influenza A virus H5N1 challenge in mice and was able to escape pre-existing neutralizing antibodies against AdHu5 [12]. Similarly, a porcine adenovirus 3 (PAV3) vector was successful in several swine vaccination studies against classical swine fever and pseudorabies virus [13], [14], [15]. PAV3-based vaccines were able to evade pre-existing immunity and provide long-term protection in pigs [14]. The antigenic profile and reported *in vivo* efficacy as an animal vaccine makes the PAV3 vector a promising alternative adenovirus vector for human administration.

Due to their genetic diversity and the availability of several significantly different isolates, avian influenza H5N1 viruses provide a valuable and challenging disease model for evaluating broad immune responses generated by potential adenovirus vectors. The external hemagglutinin (HA) glycoprotein mediates receptor binding, fusion, and can generate both strong antibody and cell-mediated immune responses [16] which can be directly assayed and provide useful comparison of adenovirus platforms. Highly pathogenic avian influenza H5N1 viruses have spread throughout domestic and aquatic bird populations in South East Asia and the World Health Organization (WHO) has confirmed 500 clinical cases of H5N1 cross-transmission into humans. Despite the limited incidence of human-to-human-transmission, high mortality rates (>60%) and continuous evolution of the virus represent a concern for future influenza pandemics [17], [18]. The emergence of pandemic swine-like H1N1 influenza A virus isolates in early 2009 highlights the need to generate cross-protective and lasting immune responses against diverging human and zoonotic influenza viruses. In addition to evaluation of different adenovirus platforms, the development of improved influenza vaccines would also help in better preparation against emerging pandemic viruses and could reduce the impact of infection on public health.

This study evaluates the protective efficacy following lethal homologous challenge of a replication-incompetent porcine adenovirus 3 (PAV3) vector expressing the HA gene from the A/Hanoi/30408/2005 H5N1 (H5N1-H05) influenza A isolate (PAV3-HA). The immunogenicity of HA and the success of previous AdHu5 H5N1-HA vaccines [1], [19] suggested that avian influenza H5N1 may be a good comparative model to evaluate the efficacy of a similar PAV3 vector.

## Results

### Seroprevalence of PAV3 in pooled human Ig

Previous studies showed that PAV3 does not exhibit cross-reactivity with AdHu5 or BAV3 neutralizing antibodies [20], [21]. Additionally, PAV3 was not neutralized by the lowest dilution of 1∶4 from 50 randomly selected human sera [20] In order to further address neutralization of PAV3 by an extended number of human sera, human Ig made of pooled sera from 10,000–60,000 individuals was evaluated. AdHu5, used as a control, was neutralized at the highest dilution of 1∶160 (6.25×10^−3^ mg/ml human Ig). In contrast, neutralization of PAV3 was not detected at 1∶20 (5.0×10−2 mg/ml), the lowest dilution tested. Previous studies showed little cross-reactivity between cell-mediated immune responses against AdHu5 and PAV3 [22].

### Vector construction and humoral immune responses over time

**Figure 1:**
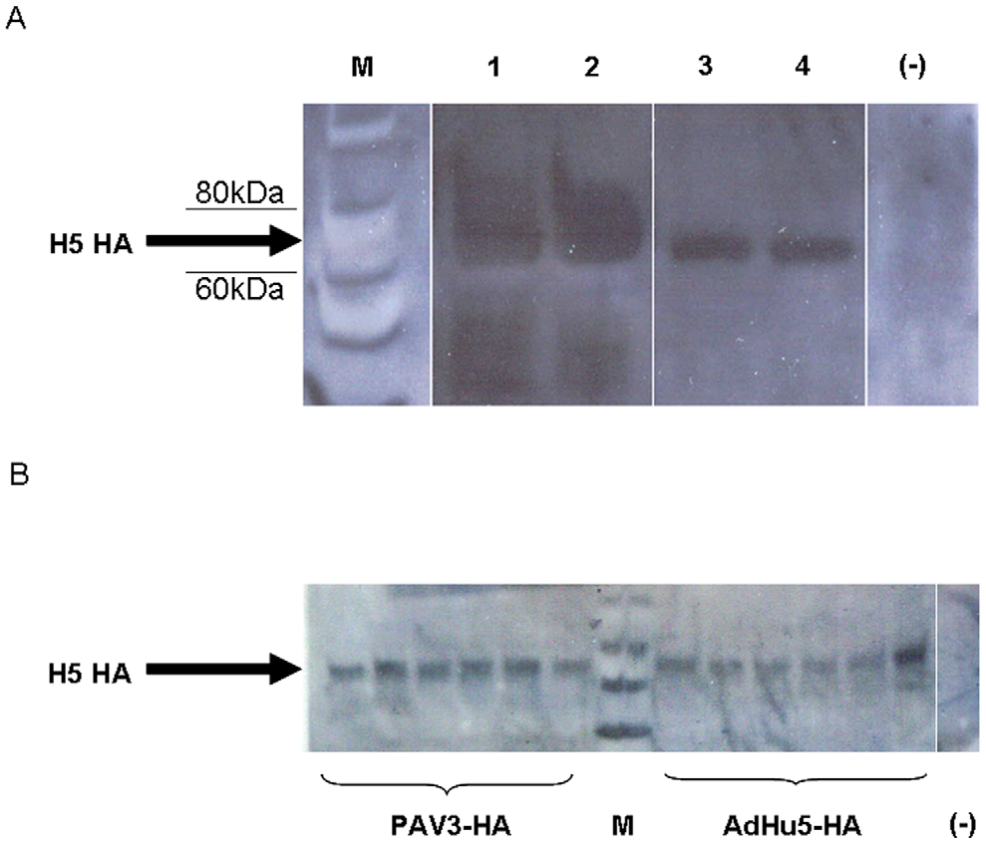
Comparative detection of H5N1-H05 HA protein expressed by AdHu5-HA and PAV3-HA. Transgene expression was detected by Western Blot using polyclonal anti-H5N1 mouse sera and a goat anti-mouse-HRP secondary antibody. (A) M = Marker, Lane 1 = expression of AdHu5-HA in HEK 293 cells, Lane 2 = expression of PAV3-HA in VRIBLE1 cells, Lanes 3 and 4 expression of AdHu5-HA and PAV3-HA in mouse syngenic AB12 cells, (−) negative control, untransfected cell lysate. (B) *In vivo* expression in mouse muscle tissues 4 days following vaccination. 75 µg of muscle tissue was loaded in each lane to compare protein expression. M = Marker, Lanes 1–6 = PAV3-HA, Lanes 8–13 = AdHu5-HA, (−) = unvaccinated muscle tissue.

**Figure 2:**
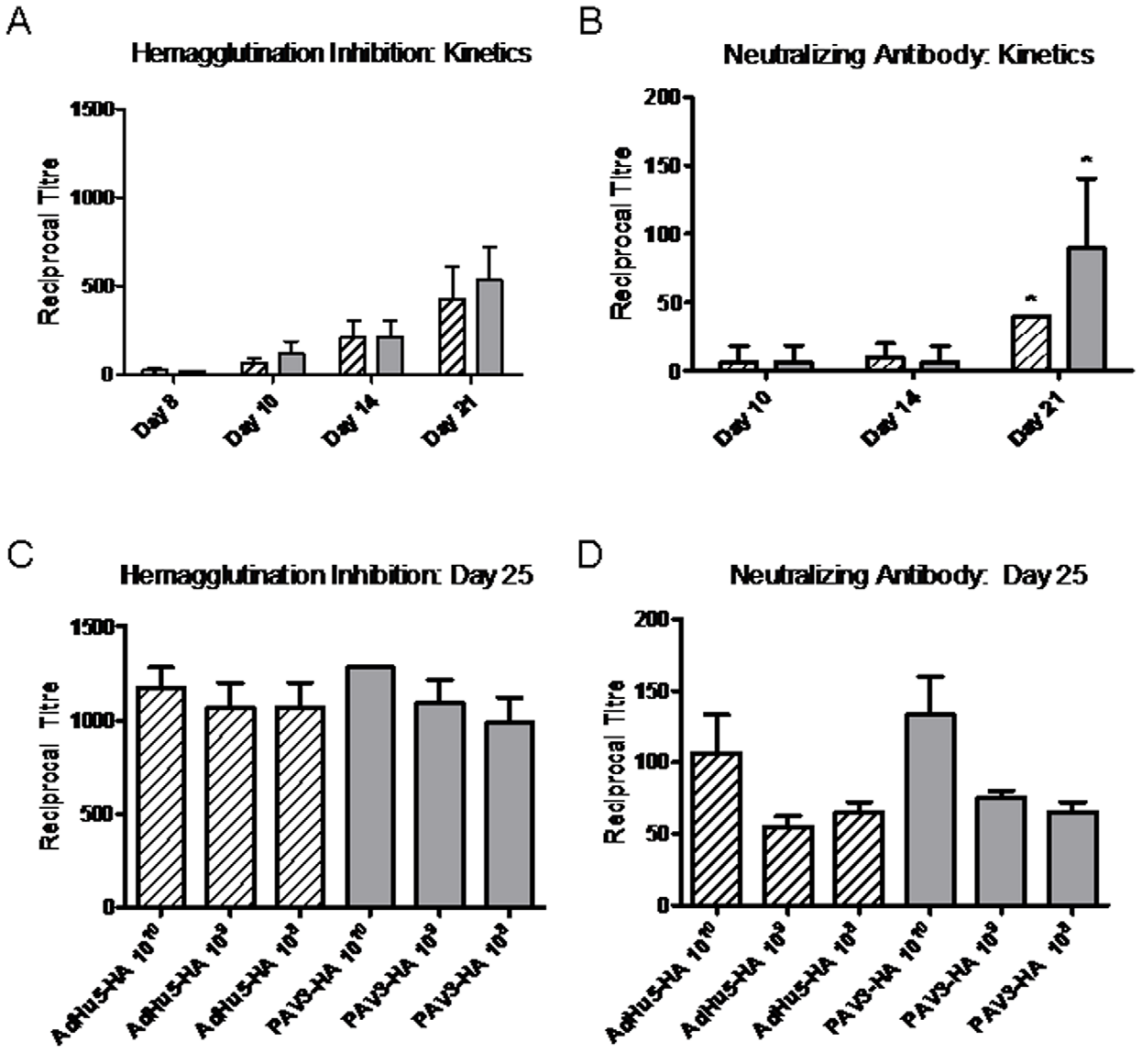
Hemagglutination inhibition (HI) and neutralizing antibody (NAB) assays for PAV3-HA and AdHu5-HA. Serum was obtained from groups of PAV3-HA (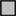) or AdHu5-HA (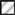) vaccinated BALB/c mice and treated overnight at receptor-destroying enzyme (RDE), followed by complement inactivation the following day. (A) Kinetics of the HI antibody response. Serum was obtained from groups of 4 BALB/c mice on days 8, 10, 14, and 21 post-vaccination with 10^10^ vp/mouse of PAV3-HA or AdHu5-HA. Four agglutinating doses of H5N1-H05 virus were added to serial dilutions of serum and samples were incubated with horse red blood cells. HI antibody titre was determined as the reciprocal of the highest serum dilution which did not cause red blood cell agglutination. (B) Kinetics of the NAB response. Serum from obtained on 8, 10, 14, and 21 days post-vaccination with 10^10^ vp/mouse of PAV3-HA or AdHu5-HA. Serial dilutions of was also incubated with 100 pfu of H5N1-H05 virus, added to MDCK cells and monitored for the presence of cytopathic effects (CPE) over 48 hours. The NAB titre was scored as the highest serum dilution with the absence of CPE. (C) HI titre against H5N1-H05. Serum was obtained 25 days post-immunization from groups of 5–10 BALB/c mice were vaccinated with 10^8^, 10^9^, and 10^10^ vp/mouse of AdHu5-HA or PAV3-HA and the HI titre was assayed. (D) NAB titres against H5N1-H05. Serum from 25 days post-immunization was also monitored for NAB titre. The data represent average values and standard deviations from 3 to 5 experiments performed with 2 independent vector preparations of each vaccine (* represents p<0.05).

An optimized expression cassette containing the codon-optimized H5N1-HA was inserted by homologous recombination into a replication-incompetent PAV3 vector containing deletions in the E1/E3 genes (described in [23]). An AdHu5-HA vaccine was also developed in parallel as a control to compare levels of protection and immune responses. Expression of the PAV3-HA and AdHu5-HA vaccines was evaluated in VRIBL E1, HEK 293, and mouse AB12 cells (Figure 1a). BALB/c mice were also vaccinated with 10^10^ virus particles (vp) and *in vivo* expression of PAV3-HA or AdHu5-HA in muscle tissue was detected 4 days post-immunization (Figure 1b). A robust antibody response is important for the prevention of influenza virus infection. The ability of the PAV3-HA vaccine to generate humoral immune responses following immunization was assayed through detection of HA-specific antibodies by hemagglutination inhibition (HI) or neutralizing antibody (NAB) titres. BALB/c mice were vaccinated with 10^10^ vp/mouse of PAV3-HA or AdHu5-HA and the development of HI and NAB antibody responses was monitored from serum collected at days 8, 10, 14, and 21 post-immunization. All samples were treated with receptor-destroying enzyme (RDE), followed by complement inactivation the next day. An HI assay was performed using serial dilutions of the treated serum combined with H5N1-H05 virus and horse red blood cells to detect inhibition of cell agglutination by serum antibody. The reciprocal of the highest dilution which did not agglutinate red blood cells was scored as the HI antibody titre. HI titres of 20±0, 120±69, 213±92, 533±184 were detected for PAV3-HA and 27±11, 67±23, 213±92, 426±184 for AdHu5-HA, respectively, with no statistical difference between the two vaccines (Figure 2a).

The presence of neutralizing antibodies to inhibit active H5N1-H05 infection was also evaluated as a measure of the humoral response. Serial dilutions of treated serum were incubated with 100 pfu/well of H5N1-H05 virus and cells were monitored for the presence or absence of cytopathic effects (CPE) using a light microscope. The highest serum dilution which did not exhibit CPE was scored as positive for neutralizing antibody and titres were reported as the reciprocal of the dilution. NAB titres of 0±0, 7±12, 7±12 and 90±50 or 0±0, 7±12, 10±10 and 40±0 reciprocal dilutions were observed with the PAV3-HA or AdHu5-HA vaccines at 8, 10, 14, and 21 days, respectively (Figure 2b). The increased neutralizing antibody response following PAV3-HA vaccination was statistically significant at day 21 (p = 0.045) relative to AdHu5-HA.

In order to assess antibody titres immediately before challenge, mice were vaccinated with different doses of each vaccine and serum was obtained 25-days post-immunization. HI reciprocal titres of 986±326, 1093±293, and 1280 for the PAV3-HA vaccine or 1066±330, 1066±330, and 1173±261 with AdHu5-HA were detected for 10^8^, 10^9^ or 10^10^ vp/mouse doses respectively (Figure 2c). Both vaccines had similar HI antibody titres at all doses, with no significant differences detected between the PAV3-HA and AdHu5-HA (p = 0.57). Based on clinical data, a reference HI antibody titre of 40 is recommended for influenza vaccine candidates as the minimum level to confer fifty percent protection in humans [24], [25]. Both vaccines met this requirement at all doses prior to challenge. Serum samples had an average NAB titre of 65±20, 75±14, or 106±46 reciprocal dilution at 10^8^, 10^9^ or 10^10^ PAV3-HA vp/mouse respectively (Figure 2d). The AdHu5-HA vaccine generated NAB titres of 65±20, 55±20 or 133±46 at 10^8^, 10^9^ or 10^10^ vp/mouse, respectively.

### Cellular immune responses over time

Although a strong antibody response is important for immediate and long-term protection against influenza viruses, the induction of early cellular immune responses following vaccination may enhance clearance of virus-infected cells following H5N1 influenza virus infection. T-cell responses were assayed using an enzyme-linked immunosorbent spot (ELISPOT) assay to detect secretion of interferon gamma (IFNγ) by activated lymphocytes. Splenocytes were obtained from mice vaccinated at days 8, 10, 14, and 21 with 10^10^ vp/mouse of PAV3-HA or AdHu5-HA and the cells were restimulated with pools of overlapping peptides corresponding to the entire H05-HA protein. The peptide corresponding to the immunodominant H5N1-H05 HA (IYSTVASSL, conserved influenza A virus) epitope was evaluated alongside an unrelated control peptide (TYQRTRALV, A/PuertoRico/8/34 H1N1 nucleoprotein).

**Figure 3:**
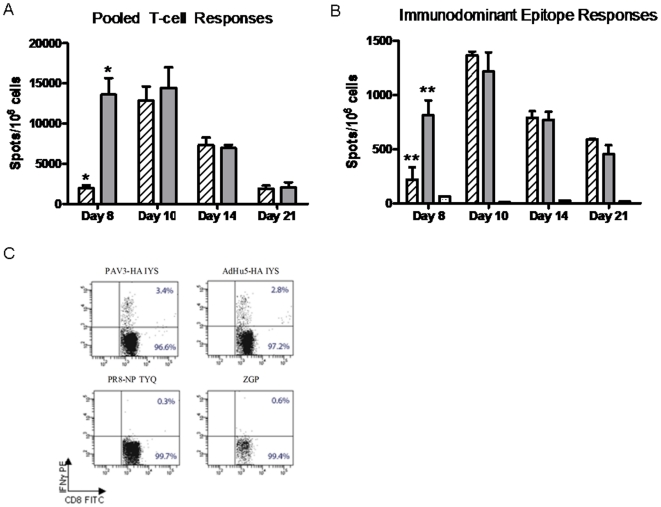
T-cell responses detected by ELISPOT-IFNγ and flow cytometry. Groups of 4 BALB/c mice were vaccinated with 10^10^ vp/mouse of either PAV3-HA (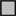) or AdHu5-HA (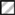). Mouse spleens were harvested 8, 10, 14, and 21 days post-immunization and positive T-cell responses following peptide restimulation were determined through IFNγ secretion, as indicated by spot formation on the membrane. (A) Pooled T-cell responses. Cells were restimulated with a peptide library of overlapping 15mers spanning the entire H05-HA protein. Bars represent the total number of spot-forming cells/million mononuclear cells for all peptide pools combined. The data represent average values and standard deviations from 4 experiments performed with 3 independent vector preparations of each vaccine (* represents p<0.005). (B) T-cell responses following stimulation with immunodominant peptide. Splenocytes were restimulated with individual 9mer or 8mer peptides representing the immunodominant epitope of H05-HA (IYS) or control peptides (PR8-NP TYQ (), ZGP). Positive responses for IFNγ secretion were detected by ELISPOT. Bars represent the total number of spot-forming cells/10^6^ mononuclear cells. The data represent average values and standard deviations from 4 experiments performed with 3 independent vector preparations of each vaccine (** represents p<0.05). C) Flow cytometric analysis representing the percentage of CD8^+^IFNγ secreting T-lymphocytes day ten post-immunization. The data represent average values and standard deviations from 3 experiments performed with one vector preparation of each vaccine.

In mice vaccinated with PAV3-HA, 13,598±2066, 14,442±2541, 6954±392, or 2056±633 spot-forming cells (sfc) per million splenocytes were detected at days 8, 10, 14, or 21 respectively (Figure 3a). Immunization with the AdHu5-HA vaccine generated 1,965±341, 12,858±1749, 7332±93, and 1902±372 sfc/million, respectively. The number of sfc/million splenocytes detected at day 8 was statistically significant between PAV3-HA and AdHu5-HA vaccines (p<0.005). The H05-HA immunodominant epitope also stimulated the strongest T-cell responses on day 8 for the PAV3-HA vaccine compared to AdHu5-HA. PAV3-HA vaccinated mice generated an average of 812±138 sfc/million at day 8 whereas 220±133 sfc/million were detected from AdHu5-HA mice at the same time point (Figure 3b, p = 0.048). At day 10, average responses following stimulation with the H05-HA immunodominant epitope were 1,216±178 or 1,364±36 sfc/million from PAV3-HA or AdHu5-HA immunized mice respectively (p = 0.258). T-cell responses of 770±74 and 453±84 were detected from PAV3-HA or 791±58 and 590±84 from AdHu5-HA at days 14 and 21 post-immunization, respectively. Re-stimulation of splenocytes from PAV3-HA or AdHu5-HA immunized mice by the NP control peptide repeatedly generated less than 65 sfc/million.

Cellular responses following vaccination with PAV3-HA were further characterized by detecting the relative frequency of peptide-specific CD8^+^ T cells expressing IFN-γ by flow cytometry. Splenocytes were harvested ten days post-immunization and re-stimulated *in vitro* with the H05-HA immunodominant peptide and unrelated NP or ZGP (TELRTFSI, *Zaire Ebolavirus* glycoprotein) control peptides. Cells were stained with anti-mouse CD8-FITC (fluorescein isothiocyanate) and an anti-mouse IFNγ-PE (phycoerythrin). The frequency of IFNγ positive CD8^+^ T-cells was 3.4±0.3 and 2.8±0.3 percent for PAV3-HA or AdHu5-HA, respectively (Figure 3c). Cells stimulated with control peptide NP or ZGP showed frequencies of CD8^+^ IFNγ positive T-cells equal to 0.3±0.05 or 0.6±0.05 percent, respectively. These results suggest that the PAV3-based vaccine can stimulate a robust immune response in mice faster than AdHu5HA and comparable in strength.

### Protective efficacy of PAV3-HA following lethal challenge

**Figure 4:**
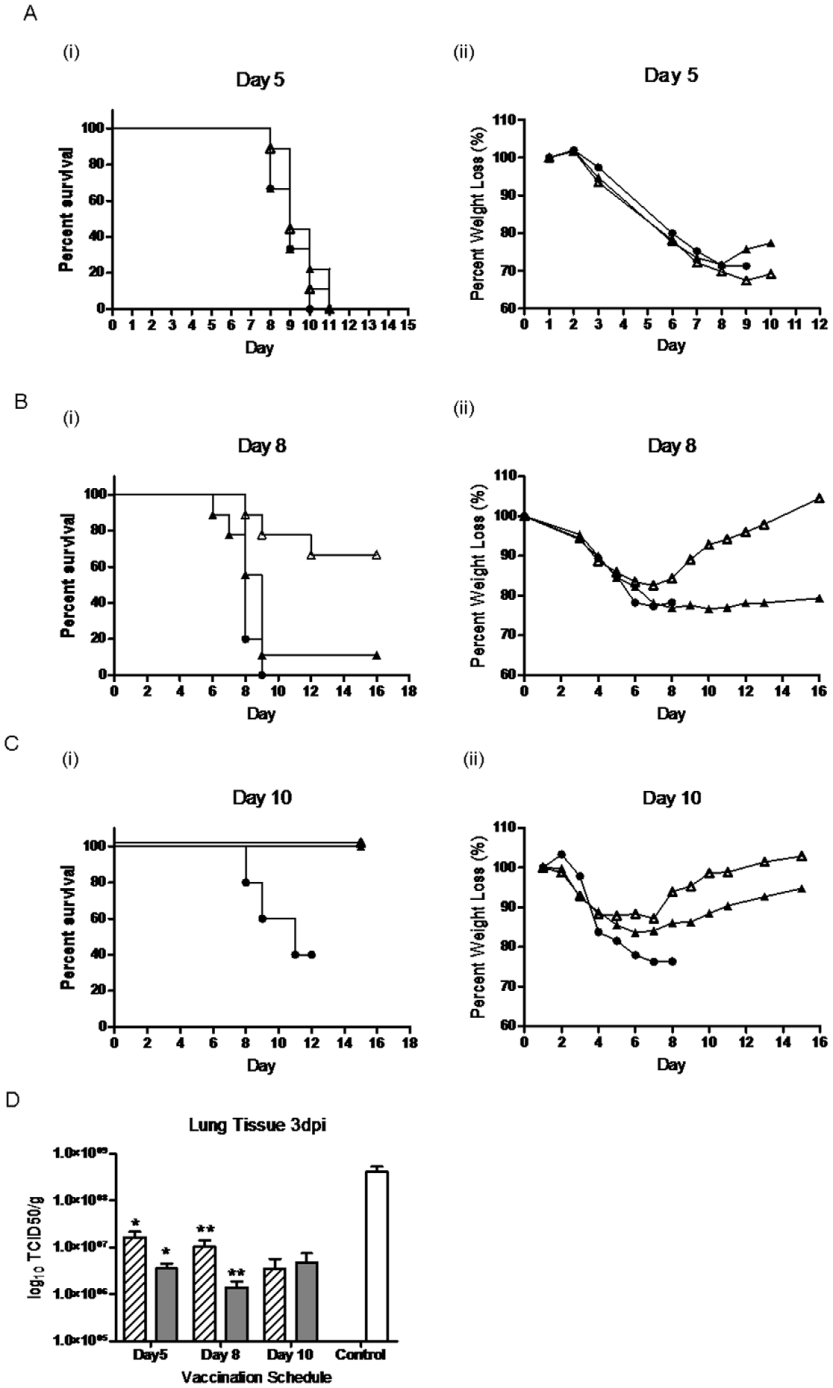
Protection following vaccination with PAV3-HA or AdHu5-HA vaccine. (A–C) Groups of 9 BALB/c mice were vaccinated with 10^10^ vp/mouse of PAV3-HA (▵), AdHu5-HA (▴), or PBS Control (•) and challenged with 100LD50 of H5N1-H05 on days 5, 8, and 10 post-vaccination. (A) Protection afforded 5-days post-vaccination. (i) survival and (ii) weight loss. (B) Protection afforded 8-days post-vaccination. (i) survival and (ii) weight loss. (C) Protection afforded 10-days post-vaccination. (i) survival and (ii) weight loss. The data represent average values and standard deviations from 2 experiments performed with 2 independent vector preparations of each vaccine (D) Lung viral titres 3 days after lethal challenge. Groups of 6 BALB/c were vaccinated with 10^10^ vp/mouse of PAV3-HA () or AdHu5-HA (), or no vaccine (Control, ()) and challenged with 100LD50 of H5N1-H05 on days 5, 8, and 10 post-vaccination. Lungs were harvested from the mice on day 3 post-challenge. Virus titre was determined by TCID50 assay using serial dilution of lung homogenates on MDCK cells and monitoring for the presence of CPE over 48 hours. The TCID50 titre was calculated by the Reed & Muench method [39] and normalized/gram of lung tissue. Data is presented as log_10_ TCID50/g of lung tissue. The data represent average values and standard deviations from one experiment performed with one vector preparation of each vaccine (* and ** represents p<0.05).

The early detection of the T-cell response at day 8 post-vaccination with the PAV3-HA vaccine suggest that it may be a good candidate for immediate administration just prior or following a suspected exposure to H5N1 virus. The success of rapid vaccination regimen was evaluated in two parts: first, overall survival and second, total viral load in lung tissue, as determined by TCID50 assay. BALB/c mice were challenged with a lethal dose of H5N1-H05 virus at 5, 8 and 10 days post-vaccination and lungs were harvested at day 3 post-infection. Although mice challenged 5 days post-vaccination did not survive lethal challenge (Figure 4a), animals challenged 8 days post-vaccination experienced significantly greater survival with the PAV3-HA (66% survival) vaccine than for AdHu5-HA (11% survival) and had less signs of disease and weight loss (Figure 4b, p = 0.038). Both vaccines were fully protective with minimal signs of disease when administered 10 days prior to challenge (Figure 4c). Lung viral titres were significantly lower at all time points with both vaccines compared to unvaccinated control animals (Figure 4d). Following vaccination with PAV3-HA, virus titers were 3.57×10^6^±6.26×10^6^, 1.40×10^6^±4.87×10^6^, and 4.82×10^6^±6.57×10^6^ TCID50/g, for days 5, 8, and 10 respectively and 1.64×10^7^±8.60×10^6^, 1.03×10^7^±6.9×10^6^, and 3.52×10^6^±5.76×10^6^ logTCID50/g for AdHu5-HA. The PAV3-HA vaccine was associated with significantly lower viral load in animals vaccinated at both days 5 and 8 compared to AdHu5-HA (p = 0.032 and p = 0.048, respectively). Both vaccines were similar on day 10 (p = 0.72).

**Figure 5:**
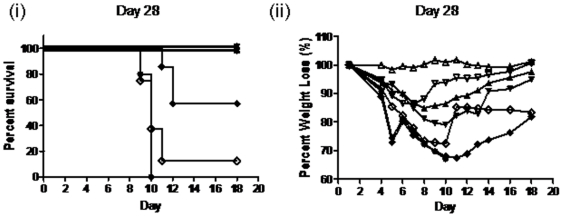
Groups of 10 BALB/c mice were vaccinated with PAV3-HA at a dose of 10^8^ (⋄), 10^9^ (▿), and 10^10^ (▵) vp/mouse, AdHu5-HA 10^8^ (♦), 10^9^ (▾), and 10^10^ (▴), or PBS Control (•). Twenty-eight days post-vaccination, the mice were challenged with 100LD50 of H5N1-H05 virus and monitored for (i) survival and (ii) weight loss and clinical signs of disease.

**Figure 6:**
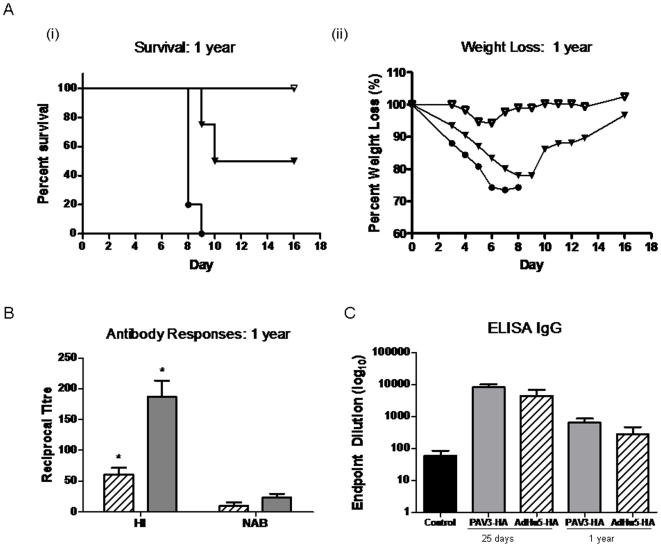
Long-term protection in BALB/c mice. Long-term protection was evaluated in groups of six BALB/c mice vaccinated with PAV3-HA 10^9^ (▿) vp/mouse, AdHu5-HA 10^9^ (▾) vp/mouse, and PBS Control (•). (A) Mice were challenged 12 months following vaccination with 100LD50 of H5N1-H05 virus and monitored for (i) survival and (ii) weight loss and clinical signs of disease. (B) Long-term antibody responses. Serum was collected 12 months post-vaccination from mice immunized with PAV3-HA (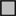) or AdHu5-HA (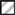). HI and NAB responses were evaluated. The data represent average values and standard deviations from one experiment performed with one vector preparation of each vaccine (* represents p = 0.006). (C) ELISA detecting total IgG antibody titres against the H5 HA antigen. Endpoint titers were assessed for PAV3-HA (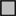) or AdHu5-HA (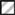) 25 days and 1 year post-vaccination.

Survival was also assessed in groups of 10 BALB/c mice vaccinated with 10^8^, 10^9^, or 10^10^ virus particles of recombinant adenovirus PAV3-HA or AdHu5-HA by intramuscular (I.M.) administration, challenged 28 days later with lethal homologous H5N1-H05 virus. Protection against clinical signs of disease with minimal weight loss and full survival was observed at the 10^10^ vp/mouse dose for both Ad-based vaccines (Figure 5). Complete survival was also observed for both vaccines at a dose of 10^9^ vp/mouse, with the PAV3-HA vaccinated mice either asymptomatic or having milder signs of disease (14% weight loss) compared to AdHu5-HA (21% weight loss). Another desirable characteristic that influenza vaccines should provide is long-term protection. Therefore, long term immunity was evaluated in mice challenged 12 months after vaccination in order to determine whether protective immune responses could be maintained. PAV3-HA afforded full protection in mice challenged with 100 LD50 of H5N1-H05 virus 12 months post-immunization whereas 50% of mice vaccinated with AdHu5-HA succumbed (Figure 6a). Higher HI antibody titers for PAV3-HA compared to AdHu5-HA (186±65 and 60±23, respectively (p = 0.006)) may have translated directly to the improved survival observed with the PAV3-HA vaccine. NAB titers were 23±15 and 10±11 for AdHu5-HA (Figure 6b). An ELISA assay was also performed to detect total IgG antibody titres against the H5N1-HA antigen. Serum was obtained from mice 25 days and 1 year post-vaccination, and unvaccinated control mice (Figure 6c). Total antibody titers were significantly lower for both vaccines after 1 year. On average, higher levels of IgG antibodies were detected for the PAV3-HA vaccine, however the difference was not statistically significant compared to AdHu5-HA (p = 0.241).

## Discussion

Currently, replication-deficient human adenovirus serotype 5 (AdHu5) vaccines are being evaluated against several pathogens. Several candidates have been shown to induce protective immune responses against emerging or re-emerging infectious pathogens such as Ebola and avian influenza (H5N1) viruses [4], [26], [27] Complete protection and long-term memory responses have also been reported in different animal models [7], [26], [28]; however, the final development of AdHu5-based vector to approved human vaccines has been hampered by the presence of natural pre-existing immunity to AdHu5 which is present in a large fraction of the human population. Neutralizing antibody to porcine adenovirus 3 (PAV3) was not detected from pooled immune globulin representing 10 to 60 thousand human sera suggesting that pre-existing immunity to PAV3-based vaccines is not likely to be of concern for human applications. In addition, the documented compatibility of porcine and human tissues could translate into PAV3-based vaccines being efficacious while of low toxicity in humans [23]. The further development of a new adenovirus serotype also increases the spectrum of possible applications such as sequential vaccination with other adenovirus vectors (e.g. prime/boost or a second Ad-based vaccination against a different agent) [20], [29].

The present study compares in parallel the immune responses and protection generated by a PAV3-based vector and a similar AdHu5-based vector using an avian influenza H5N1 disease model of infection. PAV3-based vector expressing the H5N1 HA antigen was able to generate quick and robust immune responses against H5N1. This finding was supported by the rapid induction of improved immediate protection by the PAV3-HA vaccine compared to AdHu5-HA. Interestingly, both vaccines shared similar *in vivo* expression, suggesting that the increase in vaccine efficacy observed with PAV3-HA may be due to specific interactions with the immune system rather than enhanced expression of the antigen. A recent study suggests that PAV3-HA may activate different innate immune pathways to AdHu5-HA [30]. Additionally, the PAV3-HA offered improved long-term protection against lethal H5N1 virus challenge with higher levels of detectable antibodies by HI assay. Although full short-term (28 day) survival was observed following vaccination with PAV3-HA or AdHu5-HA at the 10^9^ vp/mouse dose, greater weight loss was observed with the AdHu5-HA vaccine. Comparing HI and NAB antibody titres with observed clinical outcome, this suggests that there may have been incomplete neutralization of the virus following vaccination with AdHu5-HA, perhaps explaining why there were differences in the levels of protection observed following challenge 1-year post-vaccination. It has been shown that levels of neutralizing antibodies in serum decrease over time following H5N1 infection [31]. Following vaccination, the exact levels of neutralizing antibodies correlating with full protection from infection with homologous virus is still uncertain. In comparison with total IgG antibody levels, neutralizing antibody titres correlated better with early and long-term protection, similar to previously described reports [19], [32], [33].

Together the data suggests that PAV3 vector is an additional option to AdHu5 vector and could have several important applications including rapid or post-exposure protection against emerging influenza viruses or other infectious agents. A similar study described a BAV3-HA vaccine sharing comparable protective efficacy to the AdHu5-HA against an H5N1 virus. Two doses of the BAV3 vector generated similar humoral and cell-mediated immune responses and was able to evade pre-existing neutralizing antibodies against AdHu5 [12]. Overall protective efficacy offered by PAV3-HA was similar to AdHu5-HA one month post-immunization and the immune response kinetics was also generally comparable at later time points. Although not explored in the current study, previous studies have evaluated the impact of pre-existing immunity to PAV3 and the potential reuse of PAV3-based vectors against different pathogens. Groups of outbred pigs with high PAV3 neutralizing antibody titres were vaccinated with a PAV3-based vaccine and vector re-administration did not result in hepatotoxicity or reduced transgene expression [14]. Doses of 10^13^ particles/kg AdHu5 vector can also bypass pre-existing immunity, however, several pathologies including liver damage indicated by elevated transaminase, low platelets count, and lymphocytopenia were observed in nonhuman primates administered similar doses [9], [10], [34], [35].

Even though conventional vaccines have been relatively successful against influenza A infection, the ability of adenoviral vectors to rapidly generate a strong immune response may be useful to specific applications such as rapid immunization of health care workers in anticipation of a probable exposure to an emerging virulent pandemic virus such as H1N1-1918. In addition to strong antibody responses against the H5N1-H05 virus, both survival and cellular responses suggest that induction of an earlier T-cell response by the PAV3-may complement the developing antibody response to improve protection against H5N1 challenge and contribute directly towards lower viral load. Although the influenza glycoproteins have high antigenic variation, the generation of faster T-cell responses by a PAV3 vector against well-conserved influenza antigens may supplement a mismatched antibody response and may improve protection against a wider range of influenza viruses.

Previous studies have shown that PAV3 vectors can transduce several human cell lines resulting in full transgene expression and adenoviral coat proteins [20], [21]. An additional safety feature is that wild-type PAV3 does not replicate in human cells [21], suggesting that a replication-competent PAV3 vector which would be easy to produce at high titres could also be a promising vaccine candidate. Nevertheless, performance of this new vaccine vector will need to be addressed in other animal species, including nonhuman primates, before its real utility as a human vaccine can be predicted more accurately. Overall, this study supports a complementary role for cellular immunity during early H5N1 infection and the further development of porcine adenoviruses as human vaccine candidates.

## Materials and Methods

### Ethics Statement

All animal procedures and scoring sheets were first approved by the Animal Care Committee (Animal Use Document ID# H-08-010) at the Canadian Science Centre for Human and Animal Health, according to the guidelines set by the Canadian Council on Animal Care.

### Cells and viruses

Human embryonic kidney (HEK) 293 cells, Madin-Darby canine kidney cells (MDCK), and mouse AB12 cells were maintained in Dulbecco's modified eagle's medium (DMEM), supplemented with 10% fetal bovine serum (FBS), L-glutamate, sodium pyruvate (NaPyr), and antibiotics. Fetal porcine retina cells (VR1BL E1), expressing human adenovirus 5 (AdHu5) E1a and porcine adenovirus 3 (PAV3) E1b large genes, were maintained in minimum essential medium (MEM) alpha, supplemented with 10% FBS, L-glutamate, NaPyr, non-essential amino acids, HEPES buffered saline, penicillin/streptomycin, and 50 µg/ml Hygromycin B (BD Biosciences). Avian influenza H5N1 strain A/Hanoi/30408/2005 (H5N1) was generously provided by Q. Mai Le and T. Hien Nguyen, National Institute of Hygiene and Epidemiology, Hanoi, Vietnam. Virus was propagated on MDCK cells cultured with virus diluent (MEM, 0.3% bovine serum albumin, and antibiotics) containing 3.0 µg/ml TPCK-treated trypsin (TPCK-trypsin) and titered by plaque assay. All infectious recombinant adenovirus constructs were propagated on HEK 293 or VR1BL E1 cells and purified by a cesium chloride density gradient.

### Vectors and construction of transfer plasmids

The full complementary DNA (cDNA) sequence from the A/Hanoi/30408/2005 H5N1 hemagglutinin (H05-HA) gene was obtained, codon optimized, and synthesized from overlapping oligonucleotide primers, as previously described [36]. The H05-HA gene was first cloned into the pCAGα plasmid, containing a chicken-β-actin (CAG) promoter, to generate the pCAGα-HA construct. The pCAGα-HA expression cassette was then excised and inserted into two shuttle vector systems: pShuttle2 (Clontech) and pPAV227 (VIDO, University of Saskatchewan). Insertion of the expression cassette replaced the existing pShuttle2 cytomegalovirus (CMV) promoter with the CAG promoter, resulting in the pShuttle2-HA construct. Transfer plasmid pPAV227 was also modified to include the SV40 polyadenylation signal from pShuttle2, generating pPAV227- HA-PolyA.

The transgene cassettes from pShuttle2-HA and pPAV227-HA-PolyA were cloned into replication-deficient ΔE1ΔE3 adenoviral vectors: pAdenoX (AdHu5, Clontech) or pFPAV227 (PAV3, VIDO, University of Saskatchewan). pAdenoX-HA was generated through digestion of both pShuttle2-HA and pAdenoX by homing endonucleases I-CeuI/PI-SceI and ligation of cohesive ends using T4 DNA ligase (Invitrogen). pFPAV227-HA was generated through homologous recombination in *Escherichia coli* strain BJ5183 (*recBC*, *sbcBC*) [37] of linearized pPAV227-HA-PolyA (Eco47III/TthIII1) and pFPAV227 (PacI).

### Production of PAV3-HA and AdHu5-HA vaccine vectors

To obtain AdHu5-HA vaccine, HEK 293 cells were transfected with 10 µg of linearized pAdenoX-HA DNA in calcium phosphate (BD Biosciences) solution and cells were cultured until the appearance of cytopathic effects (CPE). Similarly, VR1BL E1 cells were transfected with 10 ug of linearized pFPAV227-HA DNA combined with Lipofectin (Invitrogen) and cells were cultured until CPE were apparent. Amplified adenoviruses containing cell lysates were harvested, freeze-thawed three times, and purified by CsCl gradients. The integrity of the H05-HA transgene cassette was confirmed through EcoRI restriction digests and by sequencing (DNA Core, National Microbiology Laboratory) with multiple primer sets. Total virus particles (vp) was determined by OD260 and total infectious particles was determined using anti-hexon antibodies against AdHu5 (AdenoX Rapid Titer kit, Clontech) or against PAV3 (VIDO, University of Saskatchewan). Four independent vector preparations were used for each vaccine. Total infectious particles and total viral particles were determined for both adenovirus vaccines, with ratios of 1∶285, 1∶333, 1∶250, and 1∶300 for PAV3-HA and 1∶150, 1∶250, 1∶220, and 1∶250 for AdHu5-HA.

### Expression of PAV3-HA and AdHu5-HA vaccines

Protein expression of H05-HA by both vectors was confirmed in HEK 293, VRIBL E1, and AB12 cells using standard Western blotting techniques. To evaluate *in vivo* expression, groups of 6 BALB/c mice were vaccinated with 10^10^ vp of PAV3-HA or AdHu5-HA and muscle tissue was harvested 4 days following immunization. Muscle tissues were homogenized and normalized per gram of muscle tissue in radioimmunoprecipitation (RIPA) buffer. Expression of each vaccine was detected from 75 µg of loaded muscle tissue.

### Immunization and viral challenge

Groups of 10 BALB/c (Charles River Canada) were vaccinated with 10^8^, 10^9^, or 10^10^ vp of recombinant adenovirus PAV3-HA or AdHu5-HA by intramuscular (I.M.) administration. Each vaccine was diluted in 100 µl and 50 µl was administered in each of the right and left hind limbs. All mice were anesthetized and challenged after 28 days through intranasal inoculation with 100 times the dose of A/Hanoi/30408/2005 virus required to obtain 50% survival (100 Lethal Dose 50 or 100LD50) in 50 µl virus diluent. The LD50 for the H5N1-H05 virus was 1.05 plaque forming units (pfu), therefore 100LD50 was 105 pfu. Mice were monitored for 15 to 20 days following challenge and signs of disease including weight loss, labored breathing, ruffled fur, and death were observed according to an approved scoring chart. All animal procedures were approved by the Institutional Animal Care Committee at the National Microbiology Laboratory (NML) at the Public Health Agency of Canada (PHAC), according to the guidelines of the Canadian Council on Animal Care. All infectious work was performed in the high biocontainment laboratory at NML/PHAC.

### Enzyme-linked immunosorbent spot assay

Groups of 4 BALB/c mice were vaccinated with 10^10^ vp of PAV3-HA or AdHu5-HA vaccines. The day before each experiment, ELISPOT-IFNγ (BD Biosciences) plates were coated with purified mouse IFNγ and incubated at 4°C, overnight. As well, overlapping 15mer peptides (Mimitopes, Australia) spanning the entire H05-HA protein were resuspended overnight in dimethyl sulfoxide (DMSO) and allocated in pools by matrix format. Spleens were harvested on days 8, 10, 14, and 21 post-immunization and splenocytes were plated at 5×10^5^ cells/well in RPMI 1640 (supplemented with 10% FBS, L-glutamine, NaPyr, HEPES, non-essential amino acids, 5×10^−3^ M 2-β-mercaptoethanol, and antibiotics) and restimulated with each of the peptide pools (2.5 µg/ml per well). An individual 9mer peptide representing the immunodominant H5N1 H05-HA (IYSTVASSL, conserved influenza A viruses) epitope was also evaluated, along with negative controls PR8-NP (TYQRTRALV, A/PuertoRico/8/34 (H1N1) nucleoprotein) and ZGP (TELRTFSI, *Zaire Ebolavirus* glycoprotein). ELISPOT-IFNγ plates were incubated at 37°C overnight (18–20 hours) and washed the following day according to the manufacturer's instructions. AEC Substrate Set (BD Biosciences) was used to develop spots formed by interferon gamma secreting cells. Spots were visualized and counted using an ELISPOT plate reader (AID ELISPOT reader, Cell Technology, Colombia, Maryland).

### Flow cytometric analysis

Splenocytes obtained on day 10 post-vaccination, plated at 2×10^6^ cells/well, were restimulated with 9mer H05-HA, PR8-NP, or 8mer ZGP individual peptides in DMEM (supplemented with 10% FBS, L-glutamine, NaPyr, HEPES, non-essential amino acids, 5×10^−3^ M β-mercaptoethanol, and antibiotics), IL2, and GolgiStop (Brefeldin A, BD Biosciences). Cells were stimulated for 5 hours and stained with anti-mouse CD8-FITC (fluorescein isothiocyanate) at 4°C for 30 minutes. BD Cytofix and permwash protocol was used to fix and permeabilized cells according to the manufacturer instructions. The following day, cells were stained for interferon gamma using anti-mouse IFNγ-PE (phycoerythrin). All samples were read on the LSRII Flow cytometer (BD Biosciences). Data was analyzed using BD FACSDiva 6.0.1 software (BD Biosciences).

### Antibody Detection

Serum for the detection of HA specific antibodies by neutralization or hemagglutination inhibition assays was collected from each mouse following immunization, as previously described [16], [38]. Briefly, all samples were treated with receptor-destroying enzyme (RDE, 1∶3 ratio, Accurate Chemical) overnight at 37°C and inactivated the following day for 45 minutes at 56°C. Starting with a 1∶10 dilution, two-fold serial dilutions of serum were done in a 96-well round-bottom microtiter plate in virus diluent (MEM, 0.3% BSA, and antibiotics), mixed with 100 pfu/well of homologous A/Hanoi/30408/2005 virus and incubated at 37°C for 60 minutes. For hemagglutination inhibition (HI) assays, two-fold serial dilutions of each sample were performed in phosphate buffered saline (PBS) and 50 µl/well was added to a 96-well V-bottom microtiter plate. Four hemagglutinating doses of homologous A/Hanoi/30408/2005 were added to each well and the plates were incubated at room temperature for one hour. Horse red blood cells (0.5% erythrocytes in 0.85% saline) were added to each well and incubated for 60 minutes. Hemagglutination titer was determined as the reciprocal of the highest dilution where red blood cells did not agglutinate. For neutralization assays, the serum/virus mixture was added onto subconfluent MDCK cells in a 96-well flat-bottom microtiter plate and incubated for 5–10 minutes at room temperature. Virus diluent supplemented with 3.0 µg/ml TPCK-trypsin was added into each well (100 µl/well) and incubated at 37°C, 5% CO_2_, for 48 hours. Cells were monitored for the presence or absence of CPE using a light microscope. The highest serum dilution which did not exhibit CPE was scored as positive for neutralizing antibody and titers were reported as the reciprocal of the dilution. All infectious *in vitro* work was performed in the high containment laboratory of NML/PHAC.

An enzyme-linked immunosorbant assay (ELISA) was performed to evaluate total IgG antibodies present in serum at 25 days and 1 year following vaccination. 96-well plates (NUNC) were coated with 1 ug/ml H5 HA antigen, overnight at 4°C. The following day, serial tenfold dilutions of serum were added to the plates and incubated for 60 minutes at room temperature. Following three washes, a goat anti-mouse conjugated to HRP was applied as the secondary antibody for 45 minutes. After an additional three washes, a 3,3′,5,5′-tetramethylbenzidine (TMB) solution was added to react with the HRP conjugate and incubated in the dark for 20 minutes. The reaction was stopped using sulphuric acid solution. The absorbance was detected at 450 nm (OD450). The endpoint cut-off (A_450_ = 0.100) was determined as the last serum dilution which was greater than two times the optical density of the negative control. Endpoint titres were reported as the reciprocal of the first serum dilution that was higher than the cut-off.

### Rapid vaccination regimen and detection of viral load

Groups of 15 BALB/c mice were vaccinated with a 10^10^ vp/mouse dose of PAV3-HA or AdHu5-HA 5, 8, or 10 days prior to challenge with 100LD50 of H5N1-H05 virus. Nine animals were monitored for survival and lung tissue and blood were harvested from the remaining 6 mice three days post-infection. Virus load was determined by the tissue culture infectious dose which will result in cytopathic effects (CPE) of fifty percent of cell culture (TCID50). TCID50 was performed by added serial dilutions of homogenized lung tissue onto MDCK cells and monitoring the presence of CPE after 48 hours. The TCID50 titre was calculated using the Reed & Muench method [39] and normalized per gram of lung tissue.

### Graphs and Statistical analysis

All graphs were generated using GraphPad Prism 5.0 and data were analyzed for statistical difference by performing unpaired t test, or one-way analysis of variance (ANOVA) when appropriate. The differences in the mean or raw values among treatment groups were considered significant when p<0.05.
